# Radio Characterization for ISM 2.4 GHz Wireless Sensor Networks for Judo Monitoring Applications

**DOI:** 10.3390/s141224004

**Published:** 2014-12-12

**Authors:** Peio Lopez-Iturri, Erik Aguirre, Leire Azpilicueta, José J. Astrain, Jesús Villadangos, Francisco Falcone

**Affiliations:** 1 Electrical and Electronic Engineering Department, Public University of Navarre, Pamplona 31006, Spain; E-Mails: peio.lopez@unavarra.es (P.L.-I.); aguirrerik@gmail.com (E.A.); leyre.azpilicueta@unavarra.es (L.A.); 2 Computer and Mathematics Engineering Department, Public University of Navarre, Pamplona 31006, Spain; E-Mails: josej.astrain@unavarra.es (J.J.A.); jesusv@unavarra.es (J.V.)

**Keywords:** judo, wireless sensor networks, ray-launching, ISM 2.4 GHz band, ZigBee, monitoring application

## Abstract

In this work, the characterization of the radio channel for ISM 2.4GHz Wireless Sensor Networks (WSNs) for judo applications is presented. The environments where judo activity is held are usually complex indoor scenarios in terms of radiopropagation due to their morphology, the presence of humans and the electromagnetic interference generated by personal portable devices, wireless microphones and other wireless systems used by the media. For the assessment of the impact that the topology and the morphology of these environments have on electromagnetic propagation, an in-house developed 3D ray-launching software has been used in this study. Time domain results as well as estimations of received power level have been obtained for the complete volume of a training venue of a local judo club's facilities with a contest area with the dimensions specified by the International Judo Federation (IJF) for international competitions. The obtained simulation results have been compared with measurements, which have been carried out deploying ZigBee-compliant XBee Pro modules at presented scenario, using approved Judogis (jacket, trousers and belt). The analysis is completed with the inclusion of an in-house human body computational model. Such analysis has allowed the design and development of an in house application devoted to monitor the practice of judo, in order to aid referee activities, training routines and to enhance spectator experience.

## 1. Introduction

Judo is one of the most popular martial arts practiced around the world. Originally developed in Japan in the late 19th century by Jigoro Kano, the practice of Judo has become popular worldwide. Judo became an Olympic sport in the 1964 Tokyo games, with active worldwide competitions being held under the auspices of the International Judo Federation (IJF), which is subdivided in five different Judo Federations around the world. The practice of Judo has been widely adopted, not only at competition level, but also as part of the physical education programs at different school levels. Judo is considered mainly a body contact sport, in which two different modalities are developed. On one hand, the technical part also known as kata, in which different techniques and movements are evaluated and on the other hand, the combat modality, called randori. In both cases, a key aspect is the postures (with a wide range of different postures and with competition scenarios at a standing level as well as floor level) and the way contact between opponents is produced. In this sense, the IJF provides assistance and guidelines for the organizers of international competitions. Following IJF rules, the environments where judo competitions are held are complex indoor scenarios in terms of radiopropagation, as some competition venues have large spectator capacity, multiple competition areas, furniture, and other facilities. Specifically, for holding international competitions, the competition venue has to have room at least for 10,000 spectators, four or five competition areas, a warming up area with a minimum of 600 m^2^, and the following services and materials must be provided for each competition area: two electronic scoreboards plus one manual, a table for at least 10 people and a smaller table for the check-in and control of judokas. In addition, a central table for a jury is needed, with a wireless microphone system connected to the public address system for use only by referees, as well as an audio-visual information system to spectators, where the information about the judokas and the results of the combats is shown. Apart from the complexity of the morphology of the environment itself, the deployed wireless systems as wireless microphones, WiFi networks, referees' headphones, wireless systems deployed to be used by the media and the huge quantity of portable personal devices that can be found within a crowded venue can make it a very complex task in terms of interference and coexistence. On the other hand, training venues are usually less complex than competition venues in terms of interferences, as they are smaller, the number of people present is much lower and there are less operating wireless communication systems, but they still have the inherent complexity of indoor scenarios in terms of radiopropagation.

In the literature, some WSN-related works for large sport venues can be found, covering topics like the development of wireless video services [1] or the design of wireless environmental monitoring system based on ZigBee for stadiums [2], but there is a need for in-depth radioplanning studies for both large (competition venue) and smaller (training venue) sport environments. In addition, wearable sensors for monitoring sport performance, training improvement and coaching support have already been developed [3–7]. In the same way, specific wireless-based applications for different sports have been also presented in the literature, like movement activity monitoring for sprinters [8], arm symmetry investigations for swimmers [9], real-time swimmer monitoring to extract stroke information [10], effort control systems [11], tracking hip angles for cyclists [12], detection of illegal race walking [13] and various general monitoring systems [14–16], but there are very few that deal with wireless-based applications in martial arts or contact sports [17,18]. Specifically for judo environments, there is no reported work about radioplanning analysis nor development of applications.

In this work, the characterization of the radiopropagation in a real judo environment for the purpose of developing potential wireless-based monitoring applications is presented. ZigBee-based XBee Pro modules operating at ISM 2.4 GHz have been used to emulate a WSN operating within a judo environment. ZigBee technology has been previously used for other sport monitoring systems [19,20]. For the purpose of analyzing this kind of environment, in Section 2, the analyzed real judo scenario is described, as well as the process of taking radiopropagation and wireless channel quality measurements. In Section 3 the description of the simulation tool used in this work, which provides accurate radioplanning data, is presented. This tool is an in-house developed 3D ray-launching software, which is completed with the inclusion of an in-house human body computational model [21]. Section 4 shows the time domain results, the estimations of received power level and Signal to Noise Ratio (SNR) values obtained by the aim of the simulation tool. These results have been compared with the measurements in order to validate them, and they have been used to analyze the feasibility of deploying ZigBee devices within judo environments. The presented analysis can aid in the optimal wireless network deployment, making the use of WSNs attractive for multiple applications in judo environments, such as monitoring vital signs [22], coaching support, anti-doping control, combat monitoring, judoka identification or helping referees (e.g., providing information about forbidden gripping or when diving head first onto the mat, which is severely sanctioned). Following this idea, in Section 5, and as the previous radiopropagation analysis allowed, the design and development of an application devoted to monitor the practice of judo is presented. Finally, in Section 6 the conclusions of the work are presented.

## 2. Experimental Section at Judo Training Venue

As the goal of this work is to study the feasibility of deploying ZigBee-based WSNs within judo environments with the aim of implementing useful and novel applications, obtaining realistic radiopropagation data within this type of environments is mandatory. For that purpose, a judo training venue with a competition-size contest area has been chosen to carry out the measurement campaign. Thus, besides analyzing a real judo training venue, an approximation to an international competition-size scenario can be studied as the tatami complies with the dimensions specified by the IJF for contest areas for international competitions (10 m × 10 m). This scenario is located in the facilities of Judo Klub Erice, a local judo club in Navarre.

**Figure 1.**
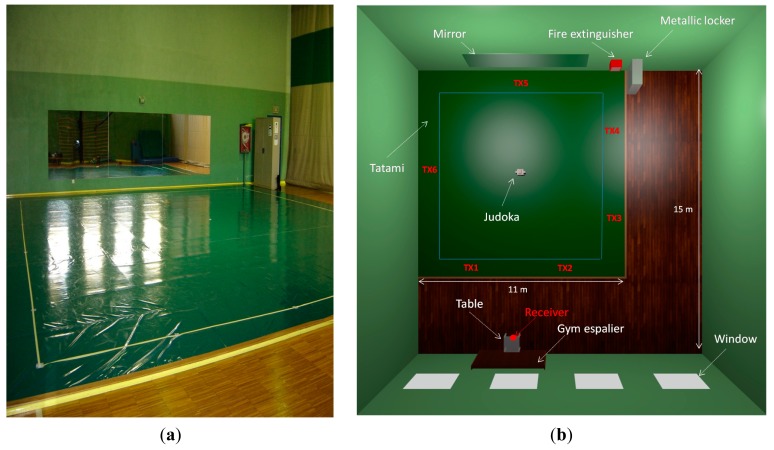
Scenario under analysis. (**a**) Real picture; and (**b**) upper view of the created scenario for 3D ray-launching simulations.

The dimensions of the whole scenario are 15 m (long) × 15 m (wide) × 8 m (height) and it has a typical morphology of a training venue, as it contains concrete walls, windows, a mirror, parquet flooring and some furniture elements as wooden gym espalier and table, metallic locker and a fire extinguisher inside a metallic box with methacrylate cover. Although most of the scenario is air, the existing furniture and the presence of people make it a complex indoor scenario in terms of radiopropagation. Figure 1 shows a picture of the real scenario where the measurements have been taken and the schematic scenario created for the later 3D ray-launching simulations, which represents the real Judo training venue.

### 2.1. Radiopropagation Measurements

In order to characterize the radiopropagation within the scenario, different measurements have been taken, firstly without and then with the presence of a judoka on the tatami. The measurements without people on the tatami will give valuable information about the behavior of the radiated electromagnetic waves at the operating frequency band (ISM 2.4 GHz) within judo environments and they will be used to validate the simulation results. The subsequent measurements with a judoka on the tatami will give more realistic data since most of the potential applications involving the practice of judo presumably include persons.

**Figure 2.**
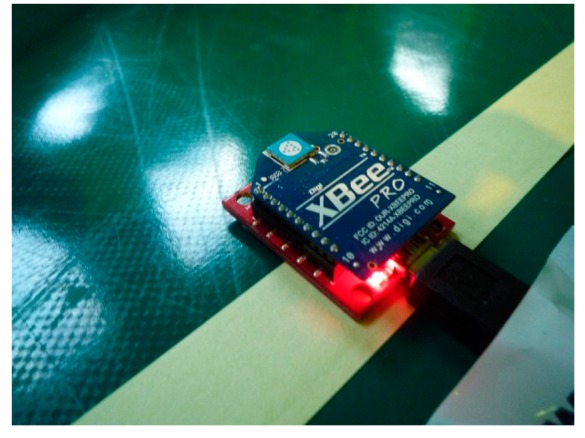
XBee Pro module deployed on the tatami.

For the setup of the measurements, a single transmitter and a receiver have been deployed. Due to the performance in terms of power consumption and spectrum availability, IEEE 802.15.4 based XBee Pro ZigBee modules with chip antenna (gain of −1.2 dBi) have been used as transmitters. For the measurements without the presence of a judoka on the tatami, and unlike the receiver, which remains in the same place, the position of the transmitter has been changed in order to cover the perimeter of the tatami. These transmitter positions are represented in Figure 1b by red text (TX) and a number. In addition, these chosen transmitter locations can emulate a potential application: by placing pressure sensors throughout the perimeter of the competition area [23,24], a wireless message could be sent to the referee's table to know if a judoka is inside or outside the competition area, which is decisive data in many occasions in order to validate a judoka's movement and award points or the combat win. Figure 2 shows an XBee Pro module deployed on the tatami. The transmission power level has been set to the maximum predefined value (18 dBm) and the transmission data rate has been set to 57,600 bps, transmitting continuously, without time interval between packets. All the measurements have been carried out statically, with the wireless motes connected to a laptop for charging as well as programming and data processing duties (e.g., PER data acquisition). The frequency of operation has been set to 2.41 GHz, which corresponds to ZigBee channel number 12 (the lowest channel allowed by these modules). The election of the frequency channel is given by the fact that possible WiFi interferences are wanted to be avoided. As a receiver, an omnidirectional antenna with 5 dBi gain, connected to a portable spectrum analyzer (Agilent N9912 Field Fox) has been used. Looking at Figure 1b again, the red point in the bottom side of the schematic representation of the scenario represents the receiver device, which has been placed on a 0.75 m height table, emulating the referees' table with a wireless node receiving the information from the environment or from the judoka on the tatami (e.g., vital signs during training sessions).

**Figure 3.**
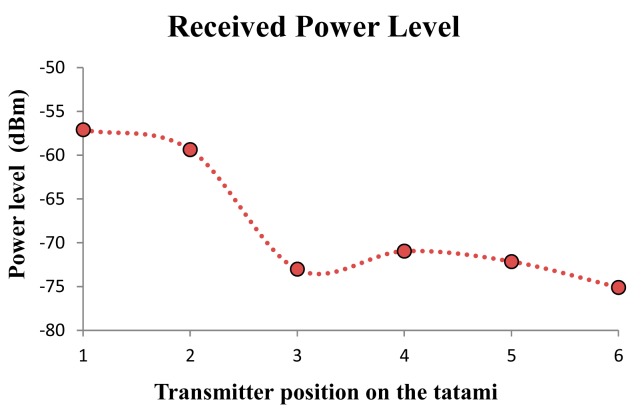
Received power level for the six different transmitter positions.

The measured power levels at receiver position for each of the mentioned six different positions of the transmitter along the perimeter of the tatami are shown in Figure 3. Thus, the radiopropagation from different distances can be analyzed. As can be seen in the obtained values, the distance between the transmitter and the receiver has an important impact on the received power, as it is expected. But it can be clearly seen how in some cases more power from further points (e.g., point TX4 *versus* point TX3) is received. This is mainly due to multipath propagation, which is the most relevant radiopropagation phenomenon within indoor environments.

**Figure 4.**
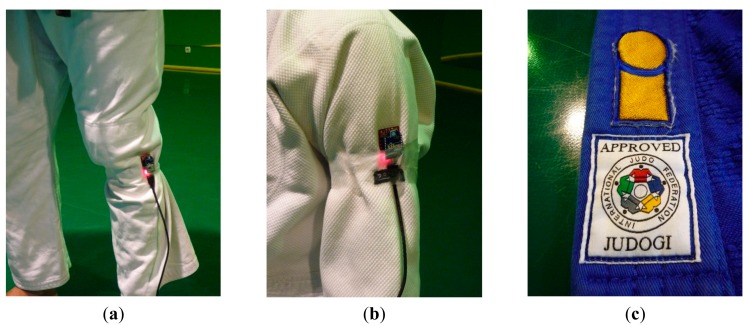
XBee Pro module on the judoka's approved judogi (**a**) on the knee; (**b**) on the arm; and (**c**) detail of the judogi's approval label.

**Figure 5.**
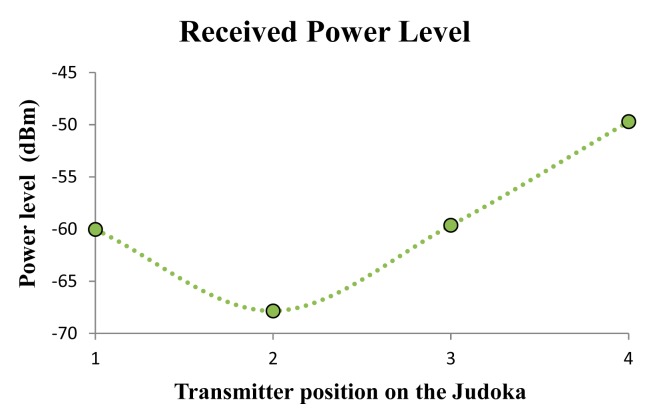
Received power level for different transmitter position on the judoka.

As it is previously stated, the judokas will be involved in the most interesting potential judo applications. Therefore, a second measurement campaign has been carried out with the presence of a judoka. The judoka has been placed in the middle of the tatami, facing the receiver point. Like in the previous measurements, the receiver is placed at the same position and it has not been moved. On the other hand, the XBee Pro transmitter has been placed at different parts of the judoka's body (chest, back, knee and arm), fixed as seen in Figure 4a,b. The motes have been configured as in the previous measurements. It is worth noting that all the measurements have been taken using two different judogis (jacket, trousers and belt) approved by IJF (see Figure 4c), in order to make them as close as possible to a realistic high level judo environment. Figure 5 shows the obtained power level values at the receiver location. As can be seen, the position of the transmitter mote on the body has a big impact in the received power level, which could be given by the fact that the receiver is placed on a table at a different height comparing to the transmitter. Note how a significant attenuation appears when the human body is between the transmitter and the receiver (transmitter on the back).

### 2.2. Wireless Channel Quality Measurements

**Figure 6.**
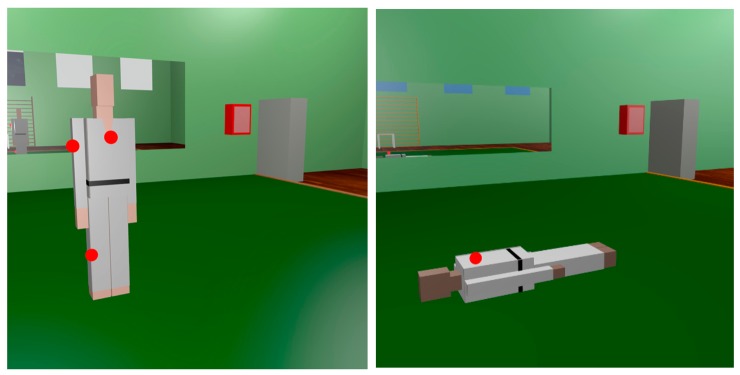
Transmitter positions (red dots) for PER measurements on standing judoka (**left**) and on lying judoka **(right**).

In addition to the radiopropagation measurements shown previously, wireless channel quality measurements have been taken in order to study if the deployed ZigBee-based communication system between a judoka/tatami and the referees' table is indeed feasible. For that purpose, a wireless link has been created between the judoka/tatami and the table with the aid of two XBee Pro modules. On one hand, the transmitter has been placed on different parts of the judoka's body, standing as well as lying in the center of the tatami (as during judo combats there are both standing and ground actions). In Figure 6 the transmitter position configurations on the judoka can be seen, where the red dots represent the XBee Pro modules. On the other hand, the same transmitter positions indicated in Figure 1b have been used for the tatami-to-table measurements. The receiver position is also the same as in the previous measurements (Figure 1b). All the measurements have been carried out with only one transmitter operating at the same time.

**Figure 7.**
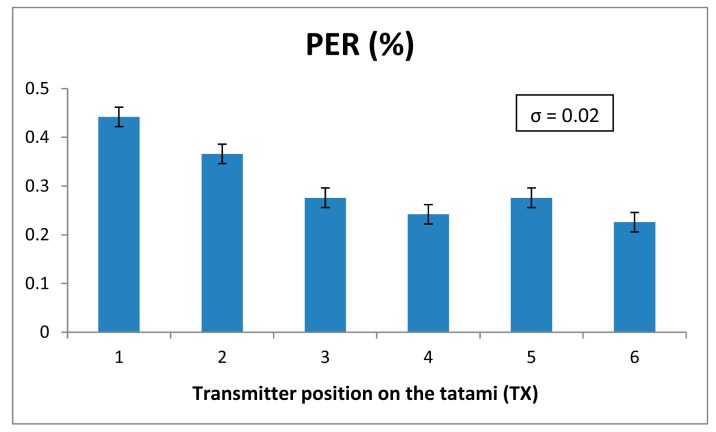
PERvalues obtained for the transmitter placed on the tatami.

**Figure 8.**
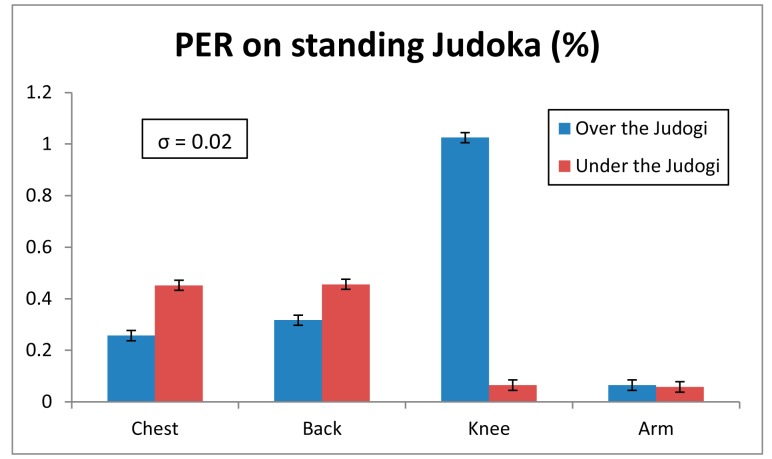
PER values obtained for the transmitter placed on a standing judoka.

**Figure 9.**
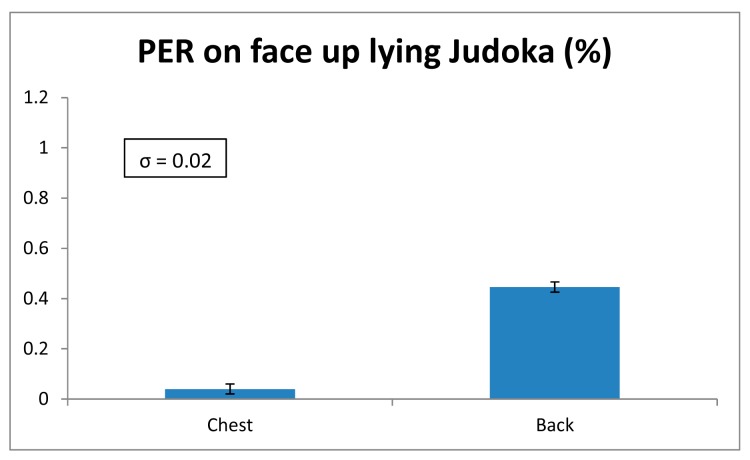
PER values obtained for the transmitter placed on a lying judoka.

The parameter chosen for the analysis of the wireless channel quality has been the Packet Error Rate (PER), which gives the percentage of the lost packets during the transmission of previously fixed packet value. In this case, 100,000 packets have been transmitted in each PER measurement. The parameters of the XBee motes have been defined like in the previous radiopropagation analysis. The transmission has been done without ACK (Acknowledgment) packets. In Figure 7 the obtained PER values for the tatami-to-table communication links are shown. Although the PER value varies depending on the position of the transmitter, this variation is very small. Besides, all the values are very low, *i.e.*, few packets are lost. The standard deviation for each measurement point is also shown, which is very low, namely 0.02% in terms of PER. This means that the PER variation is strongly dependent with the position of the transmitter. Similar results can be seen for the case with the transmitters placed on the judoka's body. In Figures 8 and 9 measured PER values for standing and lying judoka can be seen respectively. In this case, besides the fact that the PER will change depending on the position, other issues affect the transmission, like the difference transmitter position heights or the polarization of the transmitter antennas, which is the opposite for the standing judoka measurements. For a more in-depth analysis for the motes placed on the judoka, the effect of the judogi in the transmission has been analyzed. Specifically, for the standing Judoka, measurements on and under the judogi for each position have been carried out in order to quantify the PER difference. The results show that it has not great impact and the number of lost packets is still very low (see Figure 8). Finally, it is worth noting how the PER value changes when the judoka is lying face up on the tatami: comparing with the case of the mote on the chest, significantly more packets are lost when the transmitter is on the judoka's back and the body lies over the device (see Figure 9). Even so, the PER values remain low. The obtaining of these low PER values is mainly due to the sensitivity level of the XBee Pro modules, which is lower (−100 dBm) than the received power levels at the receiver position (see Figures 3 and 5 ).

From the presented channel quality measurements, and taking into account that no ACK mechanism has been used, which will significantly improve the overall performance of the wireless transmission, it can be concluded that a ZigBee-based wireless communication system can be deployed in this kind of indoor environments, since its performance is expected to be good enough for potential applications.

The next step in the analysis is to find an adequate and valid simulation method in order to obtain accurate estimations within judo environments, which will allow us to develop a complete radioplanning without the necessity of carrying out exhaustive measurement campaigns for each studied scenario. In the following section, the chosen simulation method for that purpose is presented, an in-house 3D ray launching algorithm.

## 3. Ray Launching Simulation Tool

For the assessment of the impact that the topology and the morphology of these environments have on electromagnetic propagation, an in-house developed 3D ray-launching software has been used. This simulation method offers a good trade-off between precision and required computational time for radioplanning calculations. The approach has been explained in detail in [25], and it has been validated for different complex indoor environments in [26–32].

Time domain results as well as estimations of received power have been obtained for the complete volume of the presented scenario, which have been compared with measurements in the next section. The analysis is completed with the inclusion of an in-house human body computational model, in order to understand the behavior of the propagation when a wireless mote is placed on a judoka. As far as the human body model is concerned, it has been performed with the greatest detail as possible, taking into account parts such as bones, internal organs, muscles, blood and skin, all with their respective values of dielectric constant and conductivity. The human body model has been parameterized in such a way that body proportions (*i.e*., relative dimensions between head, limbs and torso) are maintained for any given height of the person that is needed to be modeled [21].

In the ray launching method, a large set of rays is launched from the source in 3D directions with a predetermined angular separation ΔΦ (horizontal resolution) and Δθ (vertical resolution), and the rays which arrive at the receiver with a significant level of power are identified. Each ray propagates in the space as a single optic ray, with its associated electric field provided by:

(1) $$E_{i}^{⊥}=\sqrt{\frac{P_{\text{rad}}D_{t}(\theta_{t},\emptyset_{t})\eta_{0}}{2\Pi }}\frac{e^{-j\beta_{0}r}}{r}{\text{X}}^{⊥}L^{⊥}$$

(2) $$E_{i}^{∥}=\sqrt{\frac{P_{\text{rad}}D_{t}(\theta_{t},\emptyset_{t})\eta_{0}}{2\Pi }}\frac{e^{-j\beta_{0}r}}{r}{\text{X}}^{∥}L^{∥}$$

where $\beta_{0}=2\pi f_{c}\sqrt{{ɛ}_{0}\mu_{0}}$, ε_0_ = 8.854 × 10^−12^ F/m, μ_0_ = 4π × 10^−7^ H/m and η_0_ = 120π ohms. L^⊥∥^ are the path loss coefficients for each polarization ratio (*X*^⊥^, X^∥^). *P_rad_* is the radiated power and *D_t_*(*θ_t_*,∅*_t_*) the transmitter directivity.

**Figure 10.**
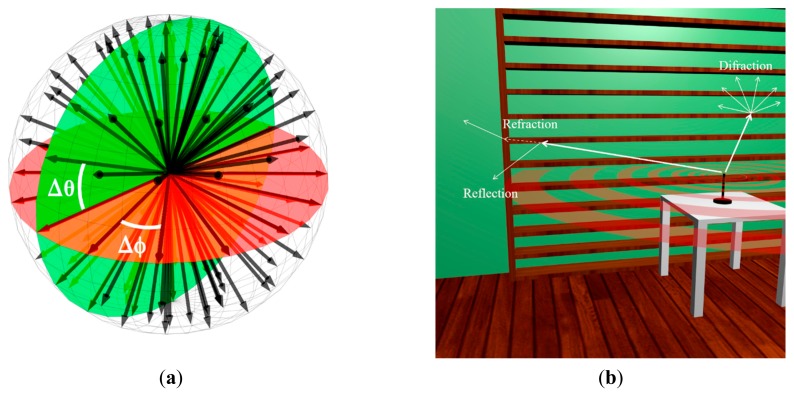
Principle of operation of the in-house developed 3D ray launching code, (**a**) angular resolution of launched rays; (**b**) electromagnetic phenomena within the scenario.

The algorithm is based on Geometrical Optics (GO) and Geometrical Theory of diffraction (GTD), with its uniform extension called as the Uniform GTD (UTD). The objective of these rays is to introduce proper field corrections, especially in the zero-field regions predicted by GO. The accuracy of the model is determined by the number of rays considered in the launched bundle and the distance to the transmitter to the receiver location. A test ray which scans a finite sample of the possible directions of the propagation from the transmitter is chosen and a ray is launched for each such direction. The number of launched rays is proportional to the dimension of the space considered. The illustration of the ray launching method is represented in Figure 10. When a ray impacts with an object, a set of a reflecting and a refracting ray is generated, and when a ray impacts a wedge, a new source of diffracted rays is created. The reflection coefficient has been computed from the classical Fresnel formula. The UTD coefficient empirically modified by Luebbers [33] has been used for diffractions.

It must be pointed out that a grid is defined in the space. When the rays are launched and then, propagate along the space interacting with the obstacles, they are causing physical phenomena such as reflection, refraction and diffraction. The principle of the algorithm is that the parameters of these rays are stored as they enter to each hexahedral until the ray has a certain number of reflections or it has exceeded the pre-propagation time set.

Parameters such as frequency of operation, number of multipath reflections, separation angle between rays, and cuboids dimension are introduced. The material properties for all the elements within the scenario are also taking into account, given the dielectric constant and permittivity at the frequency range of operation of the system under analysis.

The coefficients for the vertical and horizontal polarization for the reflected and transmitted rays are calculated according to the Fresnel equations by:

(3) $$T^{⊥}=\frac{E_{t}^{⊥}}{E_{i}^{⊥}}=\frac{2\eta_{2}\text{cos}(\psi_{i})}{\eta_{2}\cos\left(\psi_{i}\right)+\eta_{1}\text{cos}(\psi_{t})}$$

(4) $$R^{⊥}=\frac{E_{r}^{⊥}}{E_{i}^{⊥}}=\frac{\eta_{2}\cos\left(\psi_{i}\right)-\eta_{1}\cos\left(\psi_{t}\right)}{\eta_{2}\cos\left(\psi_{i}\right)+\eta_{1}\text{cos}(\psi_{t})}$$

(5) $$R^{∥}=\frac{E_{r}^{∥}}{E_{i}^{∥}}=\frac{\eta_{1}\cos\left(\psi_{i}\right)-\eta_{2}\cos\left(\psi_{t}\right)}{\eta_{1}\cos\left(\psi_{i}\right)+\eta_{2}\text{cos}(\psi_{t})}$$

(6) $$T^{∥}=\frac{E_{t}^{∥}}{E_{i}^{∥}}=\frac{2\eta_{2}\cos\left(\psi_{i}\right)}{\eta_{1}\cos\left(\psi_{i}\right)+\eta_{2}\text{cos}(\psi_{t})}$$

where $\eta_{1}=120\pi /\sqrt{{ɛ}_{r1}}$, $\eta_{2}=120\pi /\sqrt{{ɛ}_{r2}}$, and *ψ_i_, ψ_r_* and *ψ_t_* are the incident, reflected and transmitted angles respectively.

Diffraction has been implemented by edge detection in the algorithm and re-computation of equivalent scattered rays. The finite conductivity two-dimensional diffraction coefficients are given by [33] as:

(7) $$D^{∥⊥}=\frac{-e^{\left(-j\pi /4\right)}}{2n\sqrt{2\pi k}}\left\{\begin{array}{c} \cot\left(\frac{\pi +\left(\Phi_{2}-\Phi_{1}\right)}{2n}\right)F\left(kLa^{+}\left(\Phi_{2}-\Phi_{1}\right)\right)\\ +\cot\left(\frac{\pi -\left(\Phi_{2}-\Phi_{1}\right)}{2n}\right)F\left(kLa^{-}\left(\Phi_{2}-\Phi_{1}\right)\right)\\ +R_{0}^{∥⊥}\cot\left(\frac{\pi -\left(\Phi_{2}+\Phi_{1}\right)}{2n}\right)F\left(kLa^{-}\left(\Phi_{2}+\Phi_{1}\right)\right)\\ +R_{n}^{∥⊥}\cot\left(\frac{\pi +\left(\Phi_{2}+\Phi_{1}\right)}{2n}\right)F\left(kLa^{+}(\Phi_{2}+\Phi_{1})\right) \end{array}\right\}$$

where *nπ* is the wedge angle, *F, L* and $a_{-}^{+}$ are defined in [33]. *R*_0,_*_n_* are the reflection coefficients for the appropriate polarization for the 0 face or n face, respectively.

The algorithm operates in an iterative manner, considering a ray and its reflections and storing the created ray for processing later the diffraction contribution. In the last phase of the algorithm, by using the parameters stored in the previous phase and taking into account the predefined transmitter's characteristics, such as the transmitted power, the emitting antenna's directivity, the wave polarization and the carrier frequency, the relevant parameters for channel modeling are derived.

## 4. Ray-Launching Simulation Results and Discussion

In this section, the obtained simulation results are shown, and their suitability for this study is discussed. Creating a detailed scenario and as close as possible to the real one is very important in order to obtain accurate simulation results. Therefore, besides the real sizes of the elements and distances between them have been taken into account, the constitutive materials of all of the objects within the scenario have been carefully defined. A summary of the properties of the main materials used to define the constitutive elements of the created scenario are shown in Table 1.

Firstly, received power level results have been obtained for the whole volume of the scenario by means of the 3D ray launching simulation algorithm. The parameters defined for the simulations are shown in Table 2, which are equivalent to those of the used ZigBee motes.

**Figure 11.**
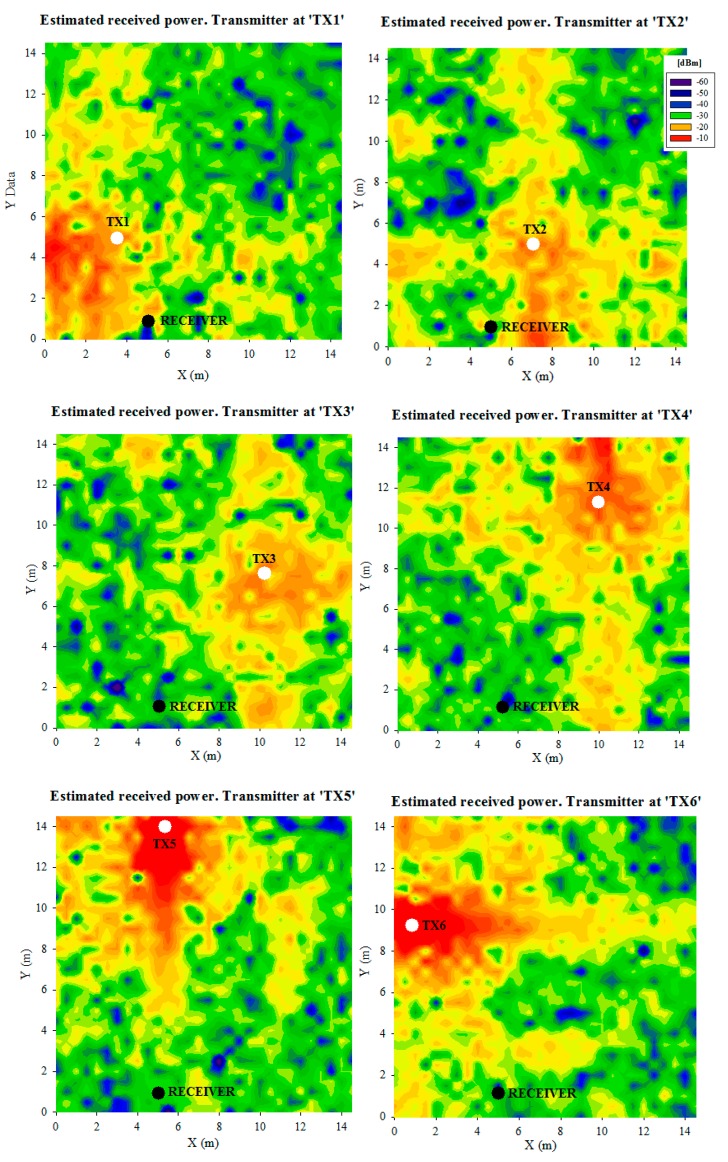
Estimated received power planes at height 0.75 m for the transmitter placed on the tatami at different positions.

**Figure 12.**
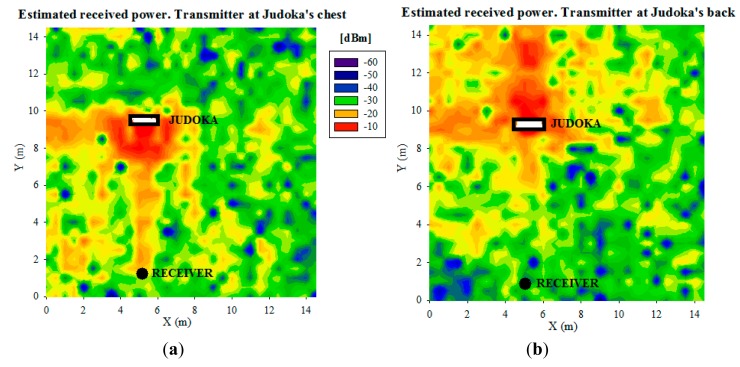
Estimated received power planes at 0.75 m for the transmitter placed (**a**) on the chest; and (**b**) on the back of a standing judoka.

Figure 11 shows the obtained received power planes at height 0.75 m (the height of the receiver on the table), when the transmitter is placed on the tatami at the six positions shown in Figure 1b, without the presence of the judoka. Figure 12 shows the same results, but for the transmitter placed on the chest and the back of a standing judoka (facing the receiver) in the center of the tatami. Note that the previously mentioned detailed human body model created in-house has been used for these simulations. As can be noticed, received power level is dependent on the position of the transmitter and receiver devices, and the influence of the human body is also clearly seen. Besides, short term variations of the received power level can be seen throughout the scenario, that are mainly due to the multipath propagation, which is in most cases the strongest phenomenon in indoor environments. In order to gain insight in the computational cost of the simulation procedure, simulation times have been obtained for different cases, which are shown in Table 3. In all cases, the simulation results have been obtained with the aid of a workstation with 2 Xeon X5650 2.66 GHz processors, 64 GB RAM and Windows XP 64 bit operating system.

**Figure 13.**
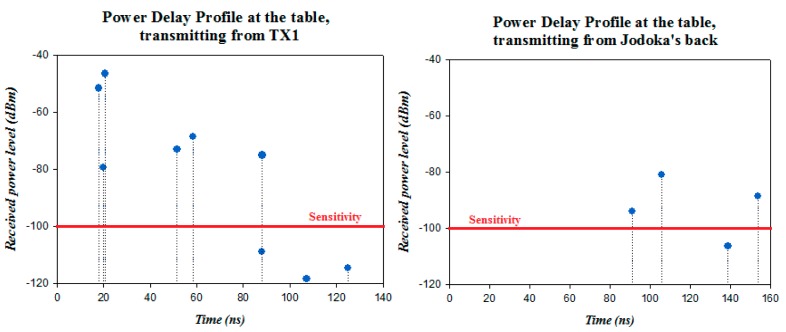
Power delay profiles at the receiver location for the transmitter placed on the tatami at position TX1 and on the back of the judoka.

In order to see the impact of the commented multipath propagation in this scenario, in Figure 13 two power delay profiles obtained from the simulation results at the center of the table are presented. The transmitter positions have been chosen in order to see the different results that can be obtained depending on the position of the transmitter. These positions correspond to TX1 and the back of the judoka. The sensitivity of the XBee Pro modules (−100 dBm) has been delimited in the graph by a red line in order to see the components which will be taken into account and which ones will not be seen by the device. The complexity of the scenario is clearly seen, as many components reach the receiver due to the number of components that are created due mainly to reflections and diffraction. As expected, more components with higher power level reach the table from position TX1 than position on the back of the judoka, as the distance is shorter and no human body is affecting the propagation.

**Figure 14.**
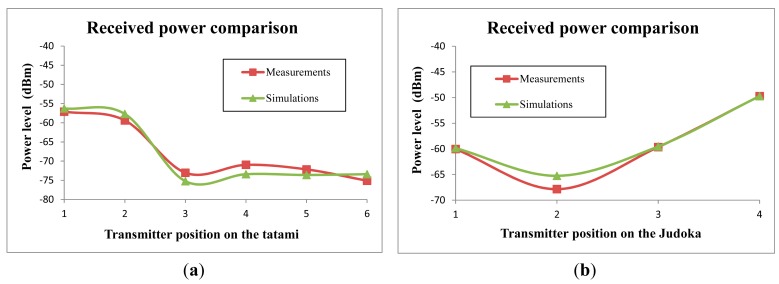
Received power comparison between simulation results and measurements for different transmitter positions, (**a**) on the tatami; (**b**) on the judoka.

In order to validate the obtained simulation results, in Figure 14 a comparison between the simulation results and the measurements of the received power level are shown. As can be seen, the received power estimations obtained by means of this method are accurate. The obtained mean error is 0.104 dB, with a standard deviation of 1.61 dB. Therefore, very accurate estimations can be obtained by means of the presented simulation method, validating it for further simulations in this kind of Judo environments.

**Figure 15.**
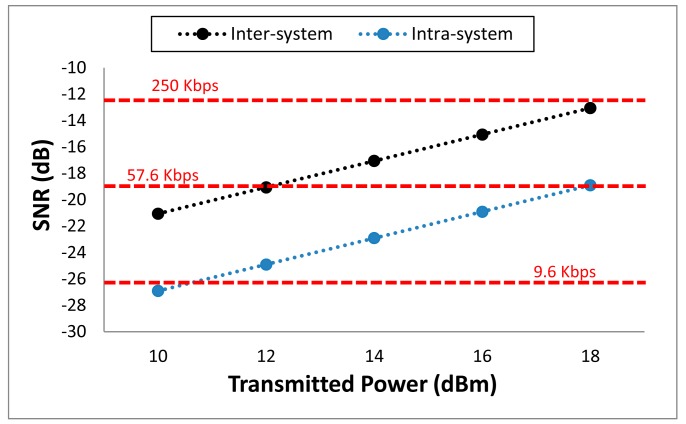
Estimated SNR values for the transmitting module on the back of the Judoka.

From the estimated received power values, link quality data can be obtained. Specifically, the SNR can be calculated at every point of the scenario. In Figure 15, as an illustrative example, a particular SNR study at receiver point for the transmitter on the back of the judoka is shown. This is the worst of the simulated cases as it is the lowest received power level. The values of the X-label are the predefined transmission power levels of the XBee Pro devices, so the SNR for each of these levels is depicted in the graph, both for intra-system (blue dots) and inter-system (black dots) interference. For the intra-system interference study, the power transmitted by the motes on the other parts of the body (chest, arm and knee) has been assumed as noise. For the inter-system interference, the interference sources are the motes placed on the tatami, emulating the interference produced by another wireless application. In both cases, it has been assumed that all the motes operate at the same frequency channel. As the SNR depends on the channel band width (3 MHz for ZigBee) of the utilized wireless system and the capacity, three red lines have been depicted in the graph, indicating the minimum SNR level required to operate at different transmission data rates (9.6, 57.6 and 250 Kbps). When transmitting at 250 Kbps, the typical highest data rate for ZigBee, the minimum SNR is not reached, even transmitting at higher power level. But typically, the data rate required for ZigBee applications is lower. For example, if the data rate is lowered to 9.6 Kbps, the SNR limit is easily overcome for most of the cases. This radioplanning analysis can be extended to other noise sources like WiFi devices or personal portable devices used by the people within the scenario.

**Figure 16.**
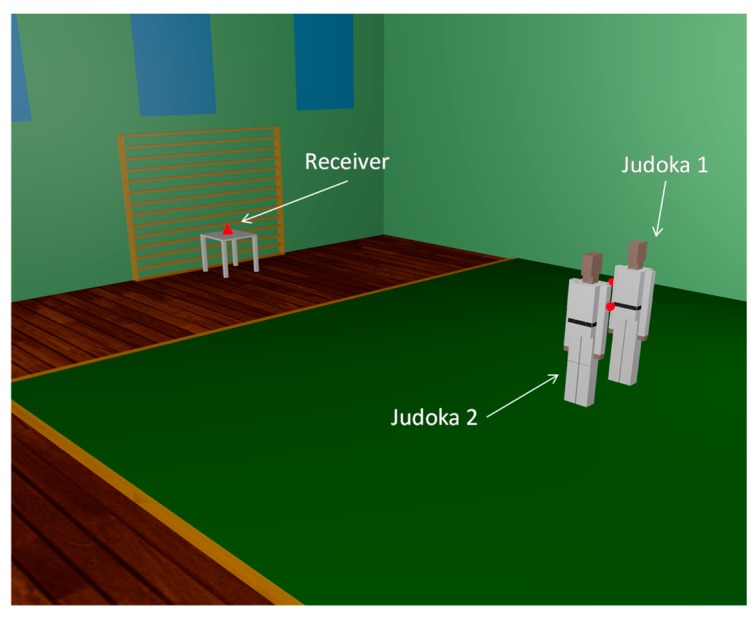
Scenario under analysis for two judokas in the center of the tatami.

**Figure 17.**
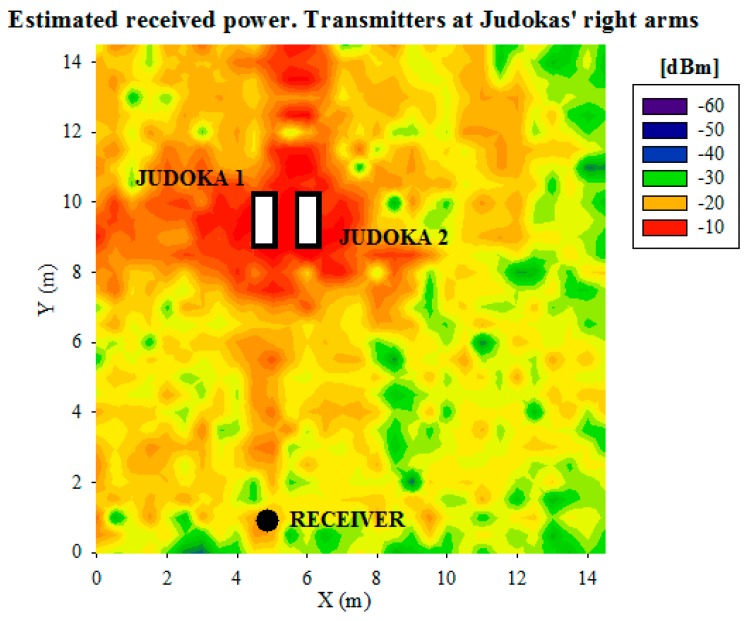
Estimated received power plane at 0.75 m for the transmitters placed on the right arm of each standing judoka.

**Figure 18.**
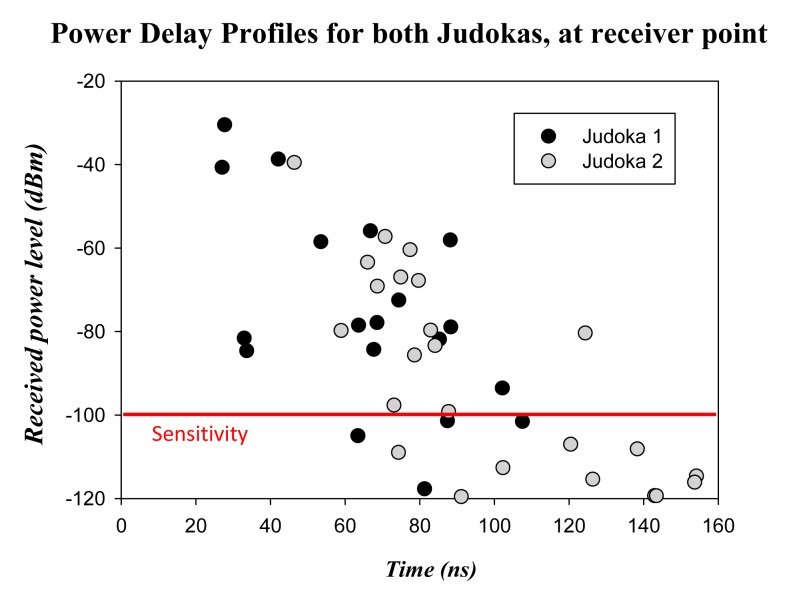
Power Delay Profiles at the receiver location for a transmitter placed on the right arm of each judoka.

Finally, once the simulation results with the presence of a judoka have been validated comparing them with measurements, further simulations have been carried out in order to analyze the effect of the presence of two judokas within the scenario, as Judo combats are played by two judokas. In Figure 16 the scenario with two judokas in pre-combat position in the center of the tatami can be seen. Each judoka has a transmitter placed on their right arm (represented by red dots). The configuration of the wireless motes (transmitters) and the parameters of the 3D ray launching algorithm are the same used for previous simulations (see Tables 1 and 2). The estimated received power for the plane at height of the receiver device (0.75 m) can be seen in Figure 17. It can be noticed how received power depends strongly on the position of the transmitters, and it can be guessed that the contribution at the received point will be different. In order to see in detail these different contributions, Figure 18 shows the Power Delay Profile at receiver point. As expected, it can be clearly seen that the rays of Judoka 1 reached the receiver with higher power level and sooner than the rays emitted by Judoka 2's transmitter. This is due mainly to the relative position of each transmitter to the receiver.

## 5. Judo Monitoring Application

Once the feasibility of deploying a ZigBee-based network in judo environments has been demonstrated, in this section an in-house developed application, based on ZigBee wireless communication is proposed. This application, devoted to monitor the practice of judo, facilitates the tasks of judo arbitration for the referee and to the two corner judges, helps the audience at competitions understand the fighting and the scores given by the referees, and allows refinement of *Judo waza* (techniques) and the training of judokas (judo practitioners). The main objective is to show in real time and with the highest accuracy, what parts of judoka's body hits the tatami. In order to obtain the maximum score, a judoka must throw his opponent on his back with impetus and control on the tatami (scoring an *ippon*). Therefore, the opponent will try to avoid falling on his back, or failing that, he will attempt to drop the front or laterally. Of course, judokas always endeavor to minimize the impact intensity. For such reason, this tool will be valid for both the arbitration of a competition, and to refine and improve judoka's training.

**Figure 19.**
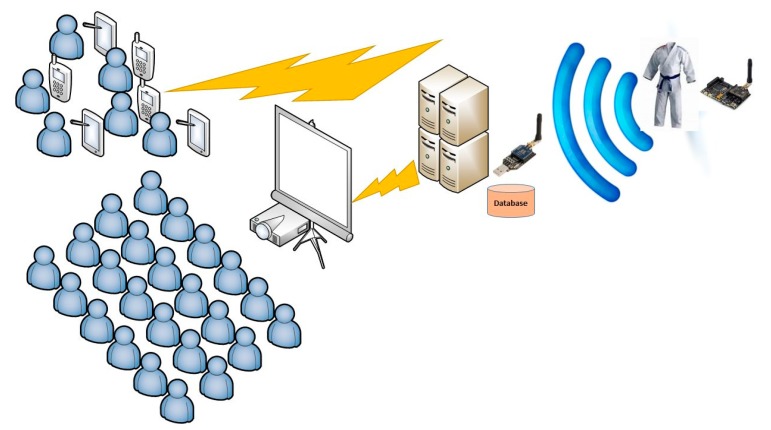
Operation schema of the application.

In the proposed application, a ZigBee mote is placed on the judogi. This mote, with the aid of embedded pressure sensors throughout the judogi, sends the amount of pressure (and its location) experimented when the judoka hits the tatami. The communication between pressure sensors and mote is wired, while communication between motes (one for each judoka) and the database wireless (ZigBee). The communication between information displays and portable devices is also wireless. Figure 19 shows schematically the architecture of the information flow from the judogi to visualization devices.

The developed application follows the Model-View-Controller (MVC) architectural pattern. The application is divided into those three interconnected parts: First, the motes collect the information from pressure sensors and transfer it in real-time to a server which is in charge of data storing. Collected data can be displayed on multiple devices, or even been stored for further analysis. Then, the controller, in this case the mote, updates the model's state with the information harvested from the pressure sensors.

The model can then interact with its associated view to change the view's presentation of the model (smartphone, TV or tablet visualization). A model notifies its associated views and controllers each change in its state, allowing the views to update their output, and the controllers to adapt to new scenarios. The output representation offered to a user depends on the type of device (smartphone, TV or tablet) and on the profile of use of the application (training, competition or refereeing).

The application is intended as a service oriented software where different technologies and languages coexist. We can distinguish three different services: data acquisition, visualization and training analysis. Those three services, powered by the server, have been developed using Java. The data acquisition service collects all the information provided by motes and stores it into a MySQL database. The sensed information is transmitted to the server by means of a ZigBee communication device. The server is a common PC with a ZigBee gateway. This information is provided to the users, previously subscribed to the visualization service by means of a WiFi multicast communication. At the client side, we have developed an Android 4.4 (KitKat) software in charge of data and the multimedia content visualization. The training analysis service allows judokas and coaches to analyze both performed techniques (*waza*) and scores obtained during a randori (judo practice). As opposite to the visualization service, the training analysis service follows a unicast TCP based communication with the server. This service allows comparing different randoris and different judokas, managing a large amount of data. It can be considered a sort of deferred telemetry. In contrast, the visualization service deals with real-time randori visualization by fans. Real time requisite imposes the use of UDP communication. Both data acquisition and training analysis are pull services, while visualization is a push service.

**Figure 20.**
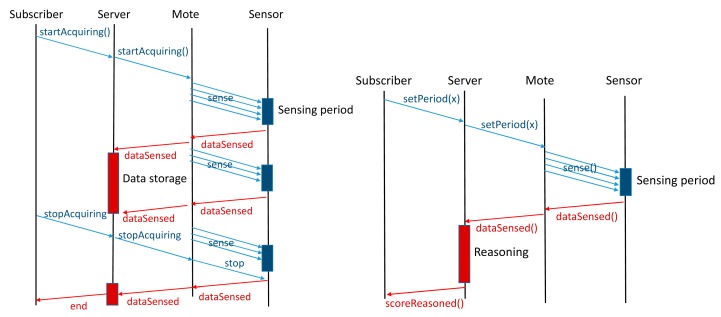
Flow diagram corresponding to data acquisition (**left**) and training services (**right**).

Visualization is a push service since, once initiated, it continuously sends the video signal (or signals, if available) to the client application (mobile application). It is a free access broadcast service to which users can freely subscribe. As opposite, data acquisition and training services are pull services due to the nature of the WSN. Subscribers select the sampling period and motes respond to the demand of the server. Both training and data acquisition services differ on the management of the information collected. The data acquisition service just stores the information collected into a database for a later processing. The training service includes the score inferred by the reasoned and allows the comparison of different stored measures. Figure 20 illustrates the sequential diagram of data transmission corresponding to data acquisition (left) and training services (right), respectively.

Motes are configured to record information using two different modes. The first one, the event driven mode, actives the network interface whenever the pressure sensor reading exceeds a certain pre-programmed threshold. This mode allows minimizing energy consumption related to communication activities, but at the cost of increasing the latency of the system. This implies the introduction of a certain delay between video signal and data processing due to the time needed to start the network interface and transmit the information sensed. This mode is used by the visualization service. The second mode attends the subscribers to determine the sampling period. The mote collects the pressure information and sends it to the subscribers of the service according to the sampling period fixed. In this mode latency is fixed by the subscribers and may be reconfigured on the fly. Subscribers pull the service on demand, selecting the sampling period. Thus applies for both acquisition and training services. The configuration as push services forces to fix the sampling period. For such reason, we chose a pull behavior of both services in order to allow on the fly customization of the sampling period by subscribers.

**Figure 21.**
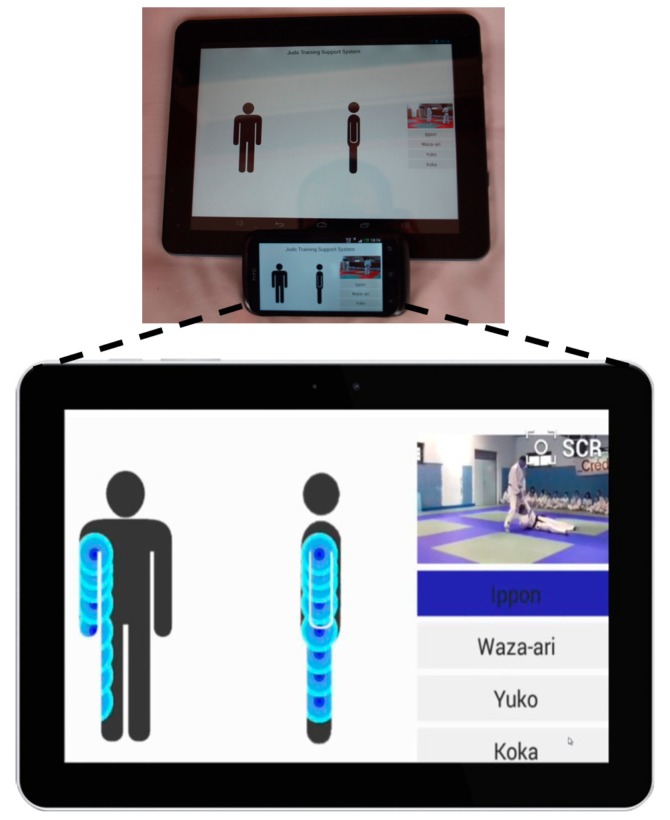
Developed application running in a tablet and a smartphone.

Figure 21 illustrates a tablet and a smartphone running the application, showing the interface provided by the visualization service, where the randori is presented at the top right of the display, while the left side of the display is devoted to represent the body areas that have impacted into the tatami and the intensity of the impact. Note that only sensed parts of the judogi (jacket and trousers) can provide information. The bottom right side of the display is devoted to display the score obtained by the execution of the technique (highlighted in dark blue).

The score obtained is inferred with the aid of a small ontology and a simple reasoner. Rules mainly concern the parts of the judogi which ones the judoka hits the ground, the total area of impact, and the intensity of the impact. The ontology has been built following the rules fixed by the International Judo Federation. For example, a *ippon* score is decided by the reasoner when the technique (*waza*) is applied correctly, or resulting to throw the opponent to fall so give his back completely on the tatami; *i.e.*, when readings of the accelerometers of the *uke*'s mote show a clean and fast path (*tori* is the person who completes the *waza* against the opponent –*uke*-) and pressure sensors measure a strong impact with a large area of the *uke*'s back. The reasoner applies the rules to these values and then infers the *ippon* score. As future works we plan to acquire by means of a camera and a pattern recognition system the scores provided by the referee of the combat. This information may allow us to better define the ontology trying to build better rules for the reasoner.

## 6. Conclusions

In this paper, the influence of judo environments in the operation of a WSN at ISM 2.4 GHz band is analyzed by means of an in-house deterministic 3D ray launching algorithm in order to aid in the development of potential monitoring applications. A campaign of measurements in a real judo training venue has been carried out in order to validate the simulation results. The results show that the topology and morphology of this kind of scenarios have a great impact on the radiopropagation and the overall performance of the WSN, due mainly to the effect of multipath propagation, which is the most significant propagation phenomenon in indoor complex scenarios. This leads to the necessity of an in-depth radioplanning tool, since the coexistence of the wireless devices of potential applications added to the portable personal devices, the presence of persons itself and other wireless systems such as WiFi networks will make it a very complex task. The high accuracy of the presented in-house 3D ray launching simulation method, including an in-house developed human body model, has been demonstrated, obtaining a mean error of 0.104 dB with a standard deviation of 1.61 dB.

Besides the feasibility of deploying a ZigBee based WSN has been demonstrated, a potential monitoring application for judo activity has been proposed and a prototype has been developed. The application covers likewise training, refereeing and entertainment needs, as it can be configured for those three user profiles and for being run in different electronic devices: smartphones, tablets and TV screens. The prototype has proved the feasibility of the service oriented design and the capacity of the communication protocols to absorb large flows of data with multiple devices and technologies operating at the same time. The feasibility of implementing an application over a ZigBee WSN and the use of precise radioplanning techniques to aid in wireless transceiver deployment can be determining factors for the adoption of these emerging technologies for Judo and other sports applications.

## Figures and Tables

**Table 1.** Main material properties in the created scenario.

**Material**	**Permittivity (F/m)**	**Conductivity (H/m)**
Wood	2.88	0.21
Plasterboard	2.02	0.06
Concrete	25	0.02
Tatami	4	0.12
Metallic	4.5	37.8 × 10^6^
Glass	6.06	0.11

**Table 2.** 3D Ray launching simulation parameters.

**Parameter**	**Value**
Transmitted power level	18 dBm
Frequency	2.41 GHz
Launched rays resolution	1°
Resolution (cuboids size)	10 cm × 10 cm × 10 cm
Maximum reflections permitted	5
Transmitter/Receiver antenna gain	−1.2 dBi/5 dBi

**Table 3.** Consumed time in 3D Ray launching simulations.

**Simulation (Transmitter Position)**	**Consumed Time (s)**
TX1	31,761
TX2	30,212
TX3	31,164
TX4	29,746
TX5	29,294
TX6	27,641
Judoka's chest	31,344
Judoka's back	30,750
